# Effects of Random Nanosized TiN Grain on Characteristic of Gate-All-Around FinFETs with Ferroelectric HZO Layer

**DOI:** 10.1186/s11671-022-03657-9

**Published:** 2022-01-21

**Authors:** Yiming Li, Min-Hui Chuang, Yu-Chin Tsai

**Affiliations:** 1 Parallel and Scientific Computing Laboratory, National Yang Ming Chiao Tung University, Hsinchu, 300 Taiwan; 2 Institute of Communications Engineering, National Yang Ming Chiao Tung University, Hsinchu, 300 Taiwan; 3 Institute of Biomedical Engineering, National Yang Ming Chiao Tung University, Hsinchu, 300 Taiwan; 4 Department of Electrical and Computer Engineering, National Yang Ming Chiao Tung University, Hsinchu, 300 Taiwan; 5 Center for mmWave Smart Radar System and Technologies, National Yang Ming Chiao Tung University, Hsinchu, 300 Taiwan

**Keywords:** Short channel effect, Gate-all-around, FinFET, Negative capacitance, Ferroelectric, Work-function fluctuation, Nano-sized metal grain

## Abstract

In this paper, we computationally study electrical characteristics for gate-all-around fin field effect transistors (GAA FinFETs) and negative capacitance GAA FinFETs (NC-GAA FinFETs) for sub-3-nm technological nodes. For the devices with the fin height of 55 nm, the on-state current increases (about 33% improvement) and the off-state current decreases (about 73% suppression) due to the NC effect. NC-GAA FinFETs have larger standard deviation of threshold voltage induced by the workfunction fluctuation (WKF) for both N-/P-type devices than those of GAA FinFETs. It is attributed to the variation of polarization in the different position of the ferroelectric layer. Notably, the inverter of NC-GAA FinFETs has larger noise margin and shorter delay time, compared with the inverter of GAA FinFETs; however, the characteristics of inverter of NC-GAA FinFETs suffer larger variability induced by the WKF.

## Introduction

In order to develop low-power and high-performance electronic devices, the conventional way is to reduce the gate length of semiconductor devices. However, the technique of scaling down has caused the serious short-channel effect (SCE); and, the high leakage current was generated. The gate cannot control the channel effectively; therefore, fin-type field-effect transistors (FinFETs) have been proposed to substitute for planar MOSFETs. The contact area between gate and channel of FinFETs can be enlarged, so the channel controllability can be improved. Hence, FinFETs have been advanced in the current fabrication [1–3]. FinFETs have two different structures, bulk and silicon-on-insulator (SOI) FinFETs, respectively. SOI FinFETs have a buried oxide layer which can be used to reduce parasitic capacitance and improve characteristics but have a worse ability of heat dissipation. After the 1st generation FinFETs, to accommodate more transistors in the integrated circuits, it is necessary to reduce the fin pitch and increase the fin perimeter so that a higher total current can be obtained proportionally. Thus, 2nd and 3rd generation FinFETs have been studied [4, 5]. Intel claims that the fin height of 3rd generation FinFETs is 53 nm and the fin pitch is 34 nm in 10-nm fabrication. Semiconductor devices with the tall fin height will generate the higher current which may become the future trend of technology in the fabrication; however, the FinFET scaling still meets serious electrostatic problems and challenges. It will lead electrical characteristics to deteriorate.

To break through the bottleneck, the gate-all-around (GAA) structure where the channel is wrapped by the insulated oxide and metal gate layers has been a good candidate which can reduce the supply voltage, sustain the gate drive capability, and promise a high performance [6]. Recent results showed that characteristics of GAA structure could be enhanced sufficiently [7, 8]. Moreover, device characteristics are influenced by the different random effects in manufacturing processes. The effect of fluctuation sources for devices is always a key issue which should be considered. Researches have focused on several kinds of fluctuation sources; such as the random interface trap [9], random dopant fluctuation [10, 11], metal gate workfunction fluctuation (WKF) [12], and others. Among these fluctuation sources, the WKF is one of the crucial factors when the process of high-κ metal gate is adopted to enhance the device performance [13]. The concept of WKF is that the random distribution of the workfunction (WK) on the metal gate because the crystal orientation is unstable during the fabrication. Additionally, to achieve better electrical characteristics, the ferroelectric (FE) material has attracted the enormous attention in recent years [14, 15]. Getting deeper insight into the material physics, the FE material has the spontaneously polarized behavior and the symmetric two stable free energy valley in the energy distributed profile can be observed. The two stable spontaneous polarization states are defined as the two degenerate energy minima. By differentiating free energy with respect to the polarization for each position in the distributed profile, we can obtain the negative capacitance (NC) region from the result of polarization versus electric field [16]. The polarized FE layer exists the NC effect; thus, based on the concept of the FE polarization effect [17, 18], the dipoles in the FE layer will be switched as applying the external bias. It will improve the high leakage current and increase the on-state current (*I*_on_) simultaneously [19, 20] and will further promise to boost the amplification gain and perform better characteristics. In order to obtain the obvious NC effect, the material of HfZrO_*x*_ (HZO) successfully attracts the wide attention [21, 22]. Thus, NC field-effect transistors (NCFETs) have been considered to suppress the SCE; for example, the sub-threshold swing (SS) of NCFETs is below 60 mV/decade which will break the Boltzmann limitation in the conventional planar MOSFETs [23]. Unfortunately, the studies on GAA FinFETs with the FE stack have not been investigated yet.

In this study, we explore FinFETs with a GAA structure for sub-3-nm technological nodes to boost device characteristics. As the fin height is increased, *I*_on_ is enhanced, while the off-state current (*I*_off_) becomes worse. Thus, we further adopt a FE stack in the dielectric layer to alleviate the SCE effectively; we investigate the effect of WKF for both N-/P-type devices and its implication in inverter. This paper is organized as follows. In “Methods” section, the device configuration and simulation techniques are described in detail. In “Results and Discussion” section, the achieved results and their physical findings are discussed. Finally, we conclude this work and suggest future studies.

## Methods

### Computational Devices

**Fig. 1:**
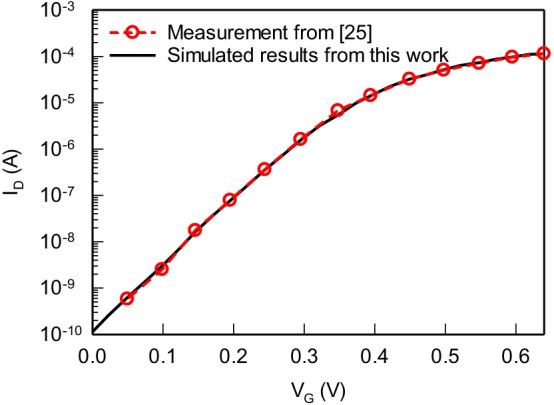
The calibrated *I*_D_–*V*_G_ curve between the simulation (black line) and the measurement (red symbol) of the N-type nanosheet MOSFET with gate length of 12 nm at *V*_D_ of 0.7 V

Based on the aforementioned devices, many researches have demonstrated the comparison between bulk and SOI FinFETs [24]. To ensure the accuracy of the following simulation, we first calibrate the *I*_D_–*V*_G_ curve with experimental data [25], as shown in Fig. 1. The calibrated silicon (Si) nanosheet device is with the gate length of 12 nm, the nanosheet thickness of 5 nm, the nanosheet width of 25 nm, the effective oxide thickness of 0.66 nm, the spacing between the channels of 10 nm, and both S and D extensions of 5 nm. The workfunction of titanium nitride (TiN), the interface trap density and the lattice temperature are tuned [26] to align the measurement.

**Fig. 2:**
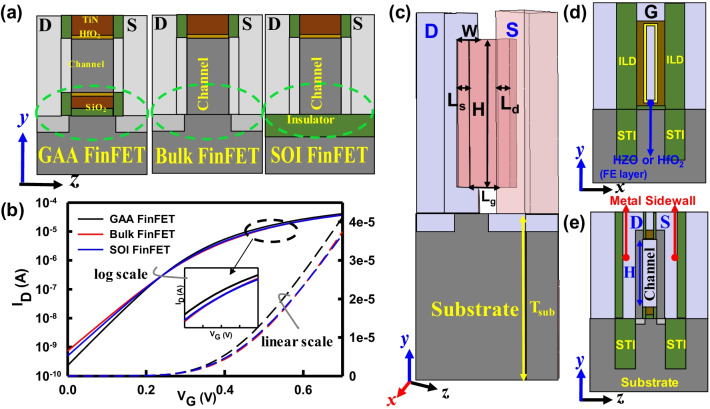
**a** The schematic plots of GAA, bulk and SOI FinFETs, respectively. **b** The *I*_D_–*V*_G_ comparison among the three structures at *V*_D_ = 0.7 V. **c** A 3D schematic of the GAA FinFET. A cross section of the 3D structure **d** in the *x–y* plane and **e** in the *z–y* plane

Figure 2 shows the schematic plots of the three Si-based structures and the corresponding *I*_D_–*V*_G_ characteristics at *V*_D_ = 0.7 V. The doping concentrations of S/D regions and channel are set as 10^20^ and 5 × 10^17^ cm^−3^, respectively. To prevent the leakage current from the S to D in the substrate, high doping concentrations (10^19^ cm^−3^) of boron and arsenic are adopted for N-/P-type FETs. As shown in Fig. 2b, the GAA FinFET has the largest *I*_on_ and the smallest *I*_off_ among these devices. Table 1 lists the extracted SCE parameters for the three devices with similar values of the threshold voltage (*V*_th_). A three dimensional (3D) schematic plot of the GAA FinFET is shown in Fig. 2c. Due to the insulated oxide and gate layers wrapping the channel, the gate can be controlled entirely; thus, devices will perform superiorly. Figure 2d, e shows cross sections of the 3D structure along different directions. In order to reduce the source/drain (S/D) resistance, the metal sidewall [26] is employed to increase *I*_on_, as shown in Fig. 2e. The inter layer dielectric (ILD) is SiO_2_ and the metal gate is TiN in Fig. 2e. Table 2 lists the adopted device parameters, where the gate length (*L*_g_) is 12 nm, the width (*W*) is 5 nm, and both source and drain extensions (*L*_s_ and *L*_d_) are fixed at 5 nm. The effective oxide thicknesses (EOT) of the device with the FE layer (w/the FE) is 0.9 nm and that of the device without the FE layer (w/o the FE) is 0.7 nm, respectively. In addition, we compare devices for the three different fin heights (*H*), i.e., 55, 70 and 85 nm, respectively. To compare the devices w/and w/o the FEs accurately, we align the similar value of *V*_th_ for all devices (*V*_th_ = 240 mV). A 3D quantum-mechanically corrected device simulation is intensively performed to assess electrical characteristics. For NC-GAA FinFETs, the material of HZO is used as the FE layer because of an easier process for HZO compared with that of the PZT [27]. The Landau–Khalatnikov (LK) equation, *E* = 2*αP* + 4*βP*^3^ + 6*γP*^5^, is solved for the FE layer [28]. The adopted HZO parameters are listed in Table 3, where *E* is the electric field, *P* is the polarization, *α*, *β* and *γ* are FE parameters which have been calibrated with the experimental data [29]. Moreover, the simulation technique of WKF mainly follows our recent work in [30]. The cuboid method can provide a robust way to predict and analyze the degree of variability accurately compared with the Voronoi method. To analyze the effect of WKF on the device characteristics, 500 devices are randomly generated by considering probabilities of 60% and 40% for the distribution of high work function fluctuation (HWKF) and low work function fluctuation (LWKF) due to the metal grain of TiN 〈200〉 and TiN 〈111〉, respectively [31, 32].

**Table 1:** Summary of electrical characteristics for GAA, bulk and SOI FinFETs, where they have the similar fin height of 20 nm, fin width of 17 nm, gate length of 22 nm, and EOT of 0.9 nm

	GAA FinFET	Bulk FinFET	SOI FinFET
*V*_th_ (mV)	240.1	240.4	240.5
*I*_off_ (pA)	230.1	733.3	493.6
*I*_on_ (mA)	41.2	37.4	36.8
DIBL (mV/V)	58.4	101.8	82.3
SS (mV/dec.)	73.0	87.9	82.3

**Table 2:** The adopted device parameters of N-/P-type GAA and NC-GAA FinFETs, where the fin heights are 55, 70 and 85 nm

Parameters	N-type devices		P-type devices	
	w/o FE	w/FE	w/o FE	w/FE
Gate length (*L*_g_)	12 nm			
Effective oxide thickness	WO/FE: 0.7 nm/W/FE: 0.9 nm			
Width (W)	5 nm			
Height (H)	55/70/85 nm			
Source extension (*L*_s_)	5 nm			
Drain extension (*L*_d_)	5 nm			
Substrate thickness (*T*_sub_)	100 nm			
Effective work function (eV)	4.437	4.377	4.908	4.939

**Table 3:** The adopted HZO parameters which have been calibrated with the measured data [28]

FE Parameter	Value
P_r_ (mC/cm^2^)	0.307
E_C_ (MV/cm)	0.185
ε_r_	16.38
*α* (cm/F)	− 5.80 × 10^10^
*β* (cm^5^/F^2^)	3.286 × 10^19^
*γ* (cm^9^/F^4^)	2.165 × 10^28^

### Settings of Workfunction Fluctuation

**Fig. 3:**
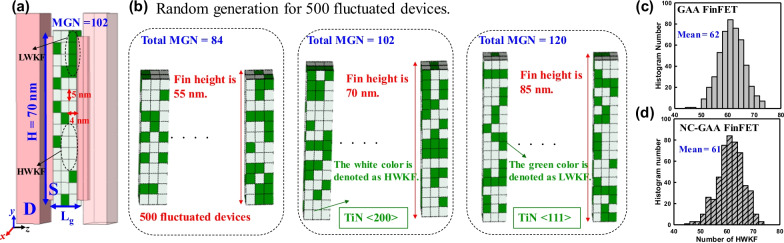
**a** The schematic plot of the device with the fin height of 70 nm which has the WK random distribution. **b** The illustrations of random generation devices with the different MGNs. Notably, the white grain color is represented as HWKF; the green grain color is denoted as LWKF. The Gaussian distributions with HWKF for **c** GAA and **d** NC-GAA FinFETs under the fixed MGNs

Figure 3a shows the schematic plot of the device with the fin height of 70 nm and a random WK distribution. Because of the variation of crystal orientation in the fabrication, the metal gate has different WK distributions. The WK of HWKF and LWKF for comparative devices is listed in Table 4. Both N-/P-type GAA FinFETs are set as the different WK and compared with NC devices in the statistical device simulation. In Fig. 3b, the different metal grain numbers (MGNs): 84, 102 and 120 which are corresponding to the fin heights: 55, 70 and 85 nm are shown. Notably, the white grain color is represented as HWKF and the green grain color is denoted as LWKF. Figure 3c, d shows the number of cases versus the number of HWKF for GAA and NC-GAA FinFETs under the fixed MGNs, where MGNs = (*L*_g_/*G*) × (*W*_eff_/*G*), *G* is the average grain size, and *W*_eff_ is the effective width of the metal gate. The randomly generated devices with a value of WK follows the Gaussian distribution.

**Table 4:** The WK of HWKF and LWKF for comparative devices

Structure name	GAA FinFET				NC-GAA FinFET			
Type	N-FET		P-FET		N-FET		P-FET	
WK function (eV)	HWKF (60%)	LWKF (40%)	HWKF (60%)	LWKF (40%)	HWKF (60%)	LWKF (40%)	HWKF (60%)	LWKF (40%)
	4.517	4.317	4.988	4.788	4.457	4.257	5.019	4.819

Both N-/P-type GAA FinFETs are set as the different WK and compared with NC devices in the statistical device simulation

## Results and Discussion

**Fig. 4:**
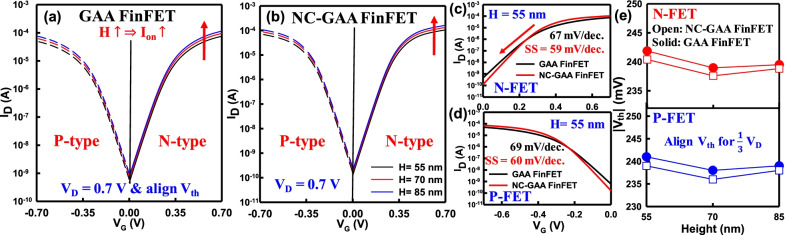
The *I*_D_–*V*_G_ characteristic of N-/P-type **a** GAA and **b** NC-GAA FinFETs with the different fin heights at *V*_D_ = 0.7 V. The curves of NC-GAA FinFETs have no hysteresis and exhibit steeper SS than that of GAA FinFETs for **c** N-/, **d** P-type devices. **e** The *V*_th_ of GAA and NC-GAA FinFETs for N-/P-type devices. To obtain the precise device simulation results, we align the similar *V*_th_ for each device under the different fin heights conditions

Figure 4a, b shows the *I*_D_–*V*_G_ characteristics of N-/P-type GAA and NC-GAA FinFETs with the different fin heights at *V*_D_ = 0.7 V. To avoid the effect of FE hysteresis [33], a thin HZO film of 2.1 nm is adopted in this simulation. We find that the *I*_D_–*V*_G_ curves of NC-GAA FinFETs exhibit steeper SS than that of GAA FinFETs in Fig. 4c, d. Figure 4e reveals the *V*_th_ for both N-/P-type devices. We extract *V*_th_ by using the constant current method: (*W*_eff_/*L*_g_) × 10^–8^ A. The values of *V*_th_ are similar by adjusting the value of WK under each fin height to obtain the physically reasonable comparison.

**Fig. 5:**
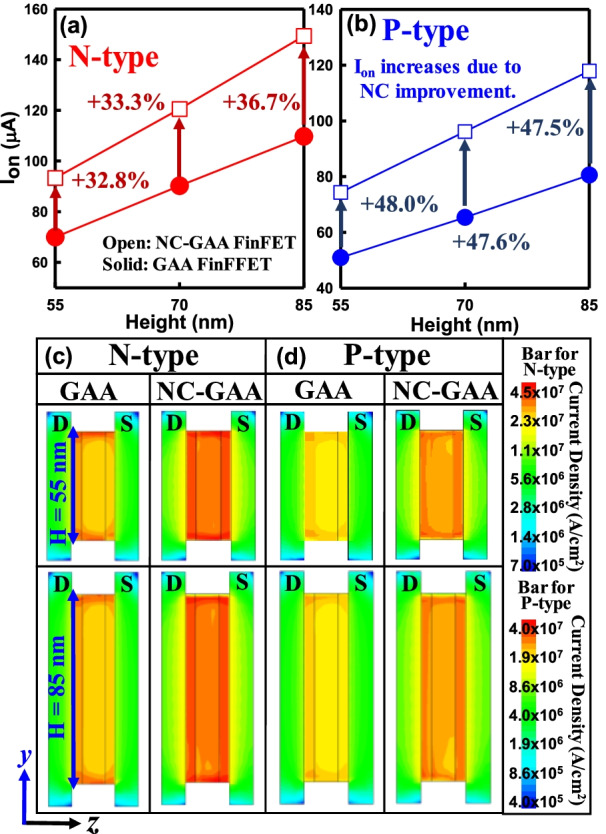
The comparison of *I*_on_ for **a** N-/, **b** P-type GAA/NC-GAA FinFETs. Because the metal sidewall can reduce the S/D resistance, the *I*_on_ is increased as the fin height increases. The current density of **c** N-/, **d** P-type GAA and NC-GAA FinFETs at the fin heights of 55 and 85 nm, respectively

**Fig. 6:**
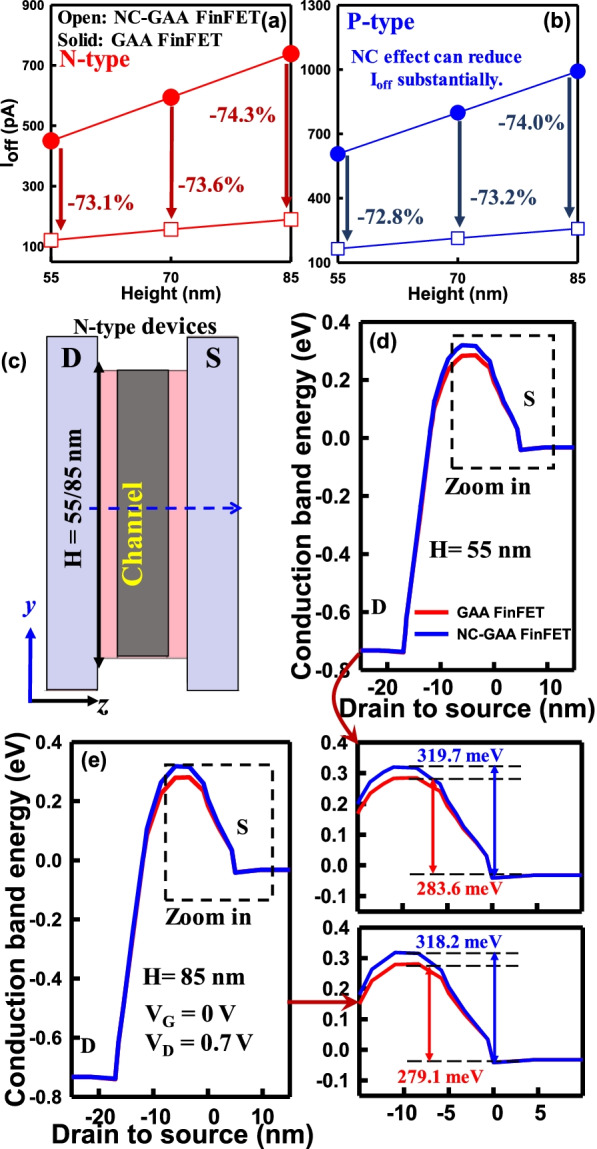
The comparison of *I*_off_ for **a** N-/, **b** P-type GAA and NC-GAA FinFETs. As the fin height increases, *I*_off_ of GAA FinFETs is enhanced. NC-GAA FinFETs improve *I*_off_ significantly. **c** The schematic plot of N-type devices which extracts the conduction band energy from D to S as the blue-dashed line. The off-state band diagram of N-type GAA and NC-GAA FinFETs with the fin heights of **d** 55 and **e** 85 nm. Compared with the GAA FinFET, the off-state band diagram of the NC-GAA FinFET shows the higher energy barrier than that of the GAA FinFET

Figure 5a, b compares the *I*_on_ for N-/P-type GAA and NC-GAA FinFETs. As the fin height increases, the *I*_on_ is enhanced because more induced charges are generated in the channel. With the fin height of 55 nm, NC-GAA FinFETs boost *I*_on_ of 32.8% for N-type and *I*_on_ of 48.0% for P-type devices compared with GAA FinFETs due to the NC effect of the FE layer. To offer a deeper insight into the mechanism of *I*_on_, as shown in Fig. 5c, d, the current density in the *z*–*y* plane for GAA and NC-GAA FinFETs with fin heights of 55 and 85 nm for both N-/P-type devices are demonstrated, which indicates that NC-GAA FinFETs can boost the current. Under the same fin height, the current density in the device w/the FE is significantly larger than that of the device w/o the FE. However, as shown in Fig. 6a, b, *I*_off_ in the device w/o the FE is increased obviously when the channel height is increased. By considering the FE layer, 73.1%- and 72.8%-reduction in *I*_off_ can be achieved for N-/P-type devices with the fin height of 55 nm, respectively. As shown in Fig. 6c, the conduction band energy from D to S (blue dashed line) will be extracted for all N-type devices. The off-state band diagrams of N-type GAA and NC-GAA FinFETs with the fin heights of 55 and 85 nm are demonstrated in Fig. 6d, e. The off-state band diagram of the NC-GAA FinFET shows a higher barrier than that of the GAA FinFET; thus, it can decrease the leakage current significantly. With the fin height of 55 nm, the off-state conduction band energy of the GAA FinFET is 283.6 meV and that of the NC-GAA FinFET is 319.7 meV which are based on the same measured point at S in the zoom-in plot of Fig. 6d; in Fig. 6e, with the fin height of 85 nm, the off-state conduction band energy of GAA FinFETs is 279.1 meV and that of NC-GAA FinFETs is 318.2 meV. The difference of off-state conduction band energy between the.

GAA and NC-GAA FinFETs with respect to the fin height of 55 and 85 nm are 36.1 and 39.1 meV, respectively. It illustrates that the variation will achieve 8.3% (((39.1 meV − 36.1 meV)/36.1 meV) × 100%) when the fin height increases from 55 to 85 nm.

**Fig. 7:**
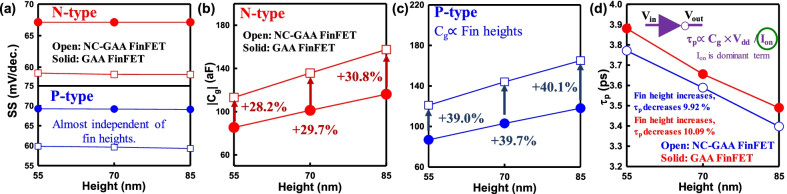
**a** A comparison of SS for N-/P-type GAA and NC-GAA FinFETs. The FE layer has the strong NC effect which causes SS lower than 60 mV/decade. A comparison of *C*_g_ for **b** N-/, **c** P-type GAA and NC-GAA FinFETs. *C*_g_ is increased as the fin height increases. **d** *τ*_P_ of GAA and NC-GAA FinFETs. *τ*_P_ of NC-GAA FinFETs is lower than that of GAA FinFETs in each fin height condition

Figure 7a demonstrates a comparison of SS for GAA and NC-GAA FinFETs with different fin heights for N-/P-type devices. The results indicate that the polarized FE layer allows SS below to 60 mV/decade. Besides, we explore the behavior of circuit applications. In order to investigate the dynamic performance of inverter for GAA and NC-GAA FinFETs, the total capacitances (*C*_g_) of N-/P- type GAA and NC-GAA FinFETs with the different fin heights are firstly shown in Fig. 7b, c. Because the channel induced charges is enhanced, the increase of *C*_g_ is proportional to the fin height. A comparison of inverters for devices w/and w/o the FE layer is revealed in Fig. 7d, where the delay time (*τ*_P_) $\propto {C}_{\mathrm{g}}\times {V}_{\mathrm{dd}}/{I}_{\mathrm{on}}$, and the *I*_on_ variation is larger than the *C*_g_ variation for both N-/P-type GAA and NC-GAA FinFETs (see Figs. 5a, b, 7b, c); thus, *I*_on_ dominates the dynamic performance of the inverter. As the fin height increases, the *τ*_P_ variation decreases ~ 10.09% for GAA FinFETs and ~ 9.92% for NC-GAA FinFETs.

**Fig. 8:**
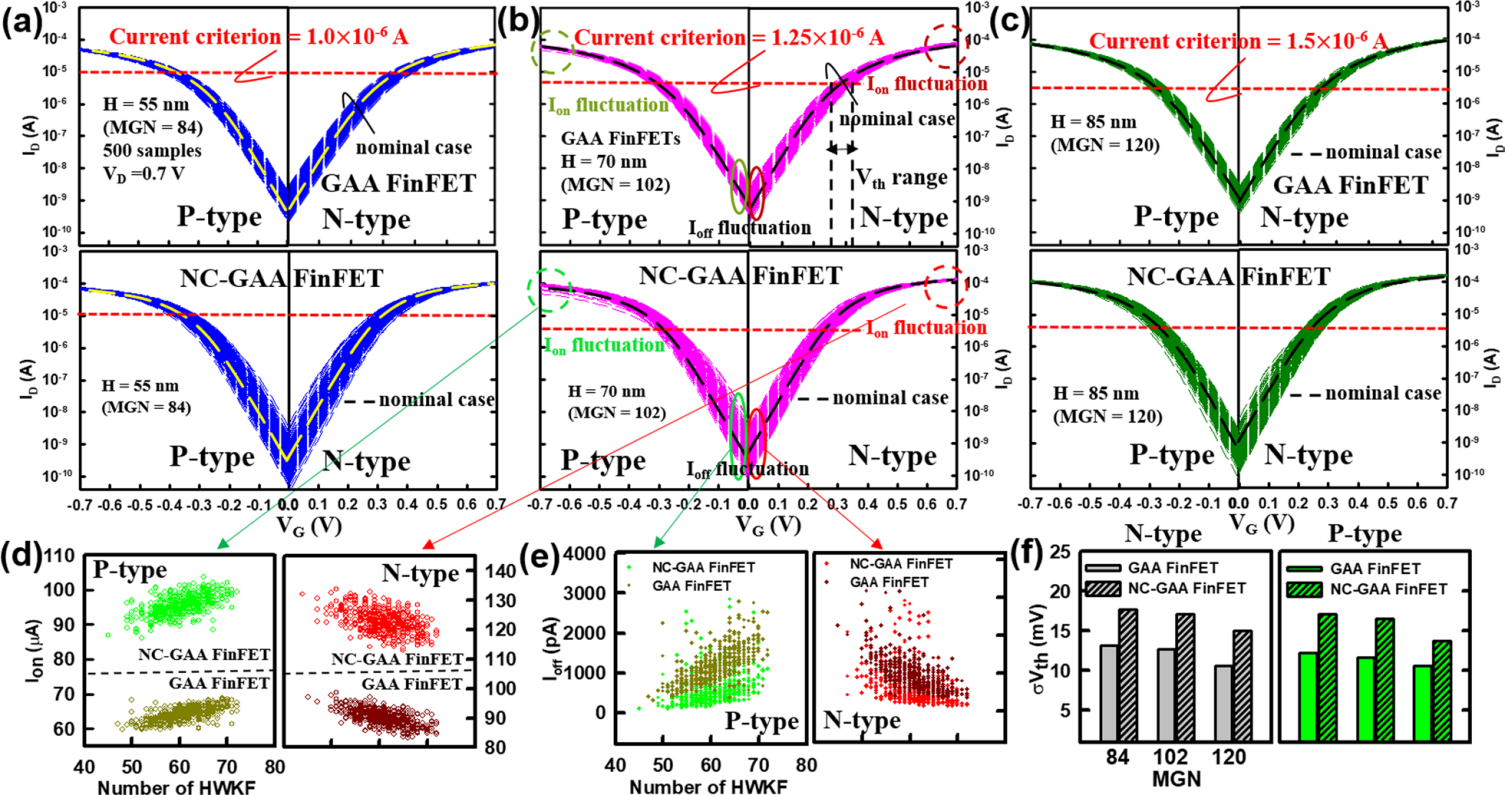
The WKF with random 500 devices for MGNs are **a** 84, **b** 102 and **c** 120. The distribution profiles of **d** *I*_on_ and **e** *I*_off_ versus HWKF numbers for N-/P-type GAA and NC-GAA FinFETs with the fixed MGN. **f** The comparison of *σV*_th_ versus the different MGNs. It can be observed that the *σV*_th_ of GAA FinFETs is smaller than that of NC-GAA FinFETs. It implies that NC-GAA FinFETs perform more sensitively induced by the WKF

Figure 8a–c demonstrates the WKF with random 500 devices for the different MGNs. Notably, the dotted lines in each figure are denoted as nominal cases. For the cases of MGN = 102 (which is corresponding to the 70-nm fin height), *I*_on_ and *I*_off_ versus HWKF numbers are shown in Fig. 8d, e, respectively. Figure 8f reveals the standard deviation of *V*_th_ (*σV*_th_) versus the different grain numbers for both N-/P-type devices. It can be observed that the *σV*_th_ of GAA FinFETs is smaller than that of NC-GAA FinFETs which indicates that NC-GAA FinFETs perform more sensitively induced by the WKF. The reason is attributed to the variation of FE polarization. Based on the concept of the FE polarization, the polarization occurs when an external bias is applied. The FE layer will suffer the different degree of the polarization in each position [16]; for example, for the nominal case of N-type NC-GAA FinFET, the polarization corresponding to close to D, the middle of channel and S are − 5.3 × 10^−7^, − 5.1 × 10^−7^ and − 5.5 × 10^–7^ C/cm^2^, respectively. Owing to the nonuniform distribution of polarization, the FE layer has significant variability induced by the WKF; thus, the effect of WKF will become seriously and *σV*_th_ will increase. It implies the usage of FE layer cannot suppress the WKF.

**Fig. 9:**
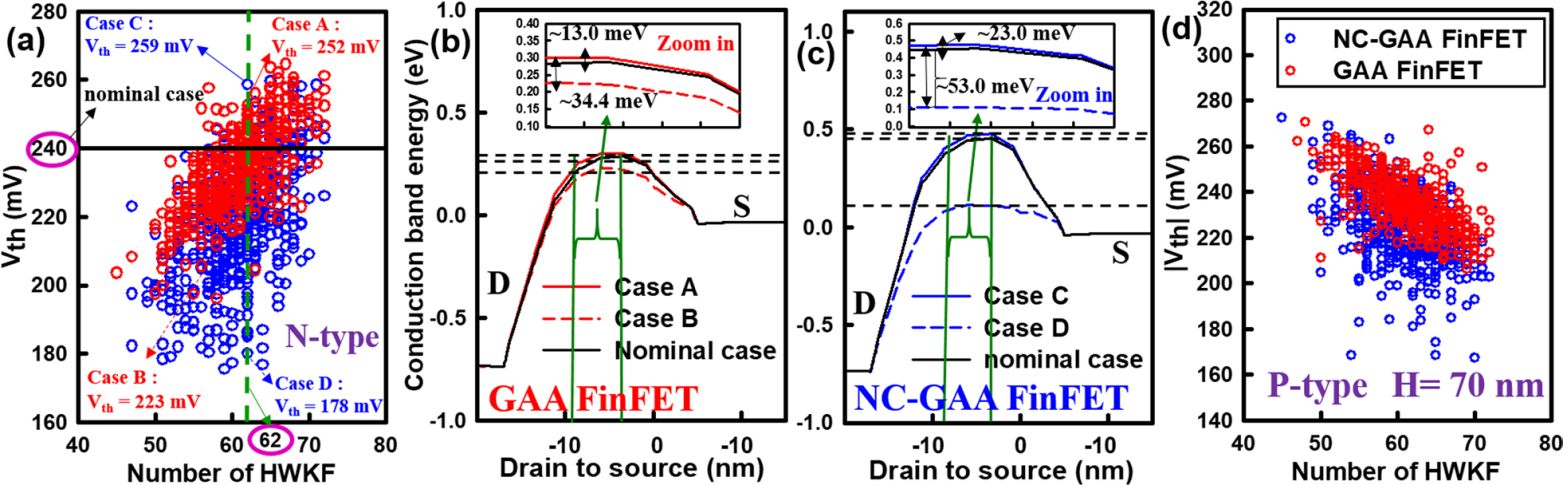
**a** The comparison of *V*_th_ distribution profile of random 500 fluctuated devices. The 1D conduction band energy profiles which are corresponding to six cases for **b** GAA and **c** NC-GAA FinFETs. **d** The relevant *V*_th_ distribution comparison profile of P-type devices

Figure 9a shows a comparison of *V*_th_ distribution of random 500 fluctuated N-type GAA and NC-GAA FinFETs. We select some cases with the same HWKF number (HWKF number is 62), such as Case A, Case B and the nominal case (*V*_th_ = 240 mV) for GAA FinFETs; Case C, Case D and the nominal case for NC-GAA FinFETs. These cases have different *V*_th_, where *V*_th_ of Case A is 252 mV, Case B is 223 mV, Case C is 259 mV, and Case D is 178 mV. The one dimensional (1D) conduction band energy profile and the zoom-in plot from D to S to demonstrate comparative curves of Case A, Case B, and the nominal case for GAA FinFETs are shown in Fig. 9b. Compared with the nominal case, Case A has a higher energy barrier, a stronger channel control ability, and a relatively larger *V*_th_. The difference of energy barrier between these cases is 13.0 meV. Similarly, the *V*_th_ of Case B is smaller than that of the nominal case which decreases 34.4 meV. The comparison of 1D conduction band profile and the zoom-in plot of Case C, Case D and the nominal case for NC-GAA FinFETs are shown in Fig. 9c. The difference of energy barrier between Case C and the nominal case is 23.0 meV; for Case D and the nominal case, the difference of energy barrier is around 53.0 meV. The *V*_th_ distribution of 500 random fluctuated P-type GAA and NC-GAA FinFETs is shown in Fig. 9d; similarly, the variation of *V*_th_ for P-type NC-GAA FinFETs is larger than that of P-type GAA FinFETs.

**Fig. 10:**
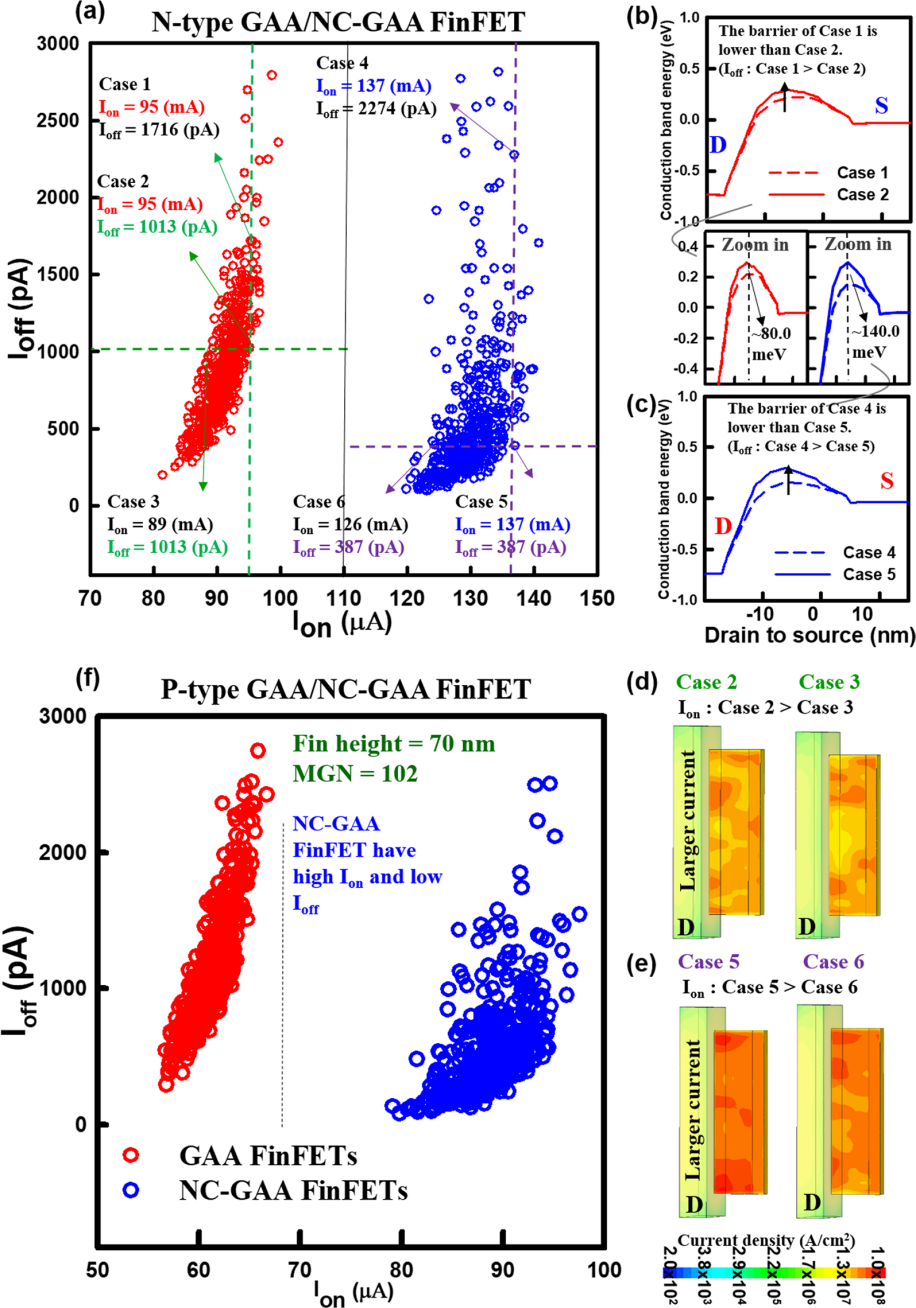
**a** The characteristic of *I*_on_–*I*_off_ for N-type GAA and NC-GAA FinFETs with the same MGN. The 1D conduction band energy profiles and the zoom-in plots for **b** Cases 1 and 2 and **c** Cases 4 and 5 for GAA and NC-GAA FinFETs. The distribution of current density for **d** Cases 2 and 3 and **e** Cases 5 and 6 for GAA and NC-GAA FinFETs, respectively. **f** The characteristic of *I*_on_–*I*_off_ for P-type GAA and NC-GAA FinFETs with the same MGN

Figure 10a shows the *I*_on_ versus *I*_off_ characteristic for N-type GAA and NC-GAA FinFETs with the same MGN. We select six cases for a further comparison. For GAA FinFETs, Case 1 and Case 2 have the same *I*_on_ but the different *I*_off_, where *I*_off_ of Case 1 is larger than that of Case 2. One-dimensional conduction band profiles and zoom-in plots of Cases 1 and 2 are shown in Fig. 10b. The energy barrier of Case 1 is 80.0-meV reduction, compared with that of Case 2 (see zoom-in plots), which leads to a larger *I*_off_. Figure 10c reveals conduction band profiles and zoom-in plots of Cases 4 and 5 for NC-GAA FinFETs with different *I*_off_. From the zoom-in plots, the energy barrier of Case 5 is higher than that of Case 4 and the difference is 140.0 meV. Figure 10d shows the distribution of the current density of Cases 2 and 3 for GAA FinFETs. A larger current density can be observed in Case 2, compared with Case 3, which leads to a larger *I*_on_. Similarly, a comparison of Cases 5 and 6 can also be investigated for NC-GAA FinFETs, as shown in Fig. 10e. Compared with GAA FinFETs for Cases 2 and 3, enhanced current densities of Cases 5 and 6 for NC-GAA FinFETs are observed obviously. *I*_on_ versus *I*_off_ characteristic for P-type GAA and NC-GAA FinFETs with the same MGN is illustrated in Fig. 10f. The trend of the distribution is similar to N-type devices; the increased *I*_on_ and decreased *I*_off_ can be obtained for NC-GAA FinFETs. Table 5 lists the standard deviation of *V*_th_, *I*_on_ and *I*_off_ for N-/P-type GAA and NC-GAA FinFETs under the same MGN; NC-GAA FinFETs have the larger variability of electrical characteristics than that of GAA FinFETs.

**Table 5:** The standard deviation of *V*_th_, *I*_on_ and *I*_off_ for N-/P-type GAA and NC-GAA FinFETs under the same MGN (MGN = 102)

	GAA FinFET		NC-GAA FinFET	
	N-FET	P-FET	N-FET	P-FET
*σV*_th_ (mV)	12.73	11.69	17.11	16.49
*σI*_on_ (μA)	2.85	1.93	3.84	3.03
*σ*log*I*_off_ (pA)	0.19	0.17	0.30	0.29

**Fig. 11:**
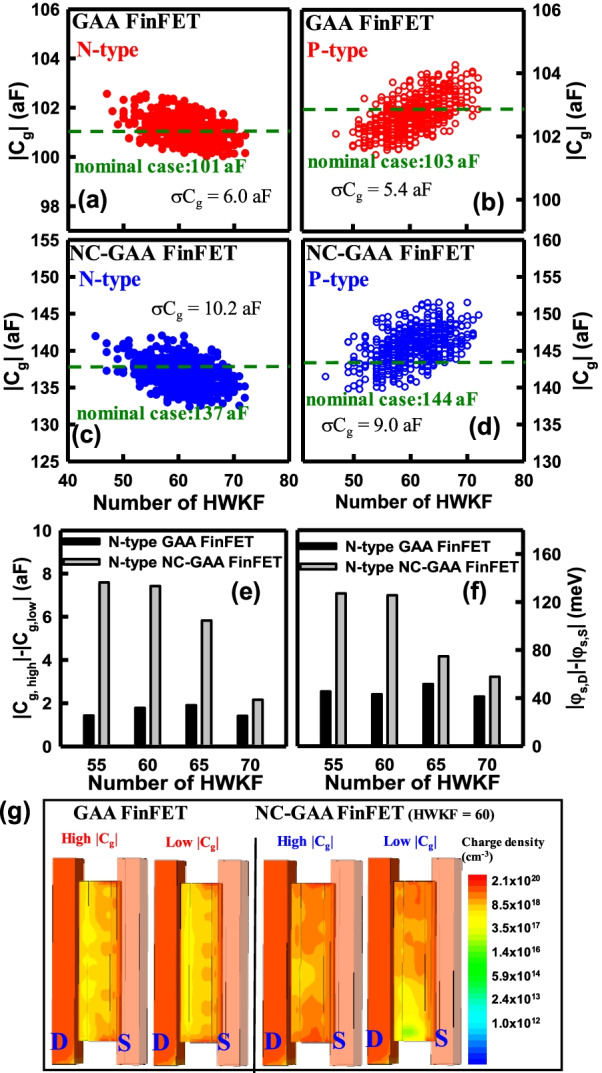
The comparison of I_on_ for **a** N-/, **b** P-type GAA/NC-GAA FinFETs. Because the metal sidewall can reduce the S/D resistance, *I*_on_ is increased as the fin height increases. The distributions of the current density of **c** N-/, **d** P-type GAA and NC-GAA FinFETs with the fin heights of 55 and 85 nm, respectively. **e** The comparison of capacitance difference (|*C*_g,high_| −|*C*_g,low_|) between N-type GAA/NC-GAA FinFETs under different HWKF numbers. **f** The surface potential (|*φ*_s,D_| −|*φ*_s,S_|) comparison of two devices under the different HWKF numbers. **g** The charge density distribution of two devices under the same HWKF number

Figure 11a, b shows the distribution of *C*_g_ versus numbers of HWKF for N-/P-type GAA FinFETs; in addition, the distribution of *C*_g_ versus numbers of HWKF for N-/P-type NC-GAA FinFETs are revealed in Fig. 11c, d. The variation of the capacitance of NC-GAA FinFETs is larger than that of GAA FinFETs, which implies that NC-GAA FinFETs have a larger variability induced by the WKF. Without loss of generality, it demonstrates 7.5% for N-type and 6.3% for P-type NC-GAA FinFETs, and 6.0% for N-type and 5.2% for P-type GAA FinFETs which can confirm that NC-GAA FinFETs will suffer the severe effect of WKF in *C*_g_. The comparison of capacitance difference (|*C*_g,high_| − |*C*_g,low_|) between N-type GAA and NC-GAA FinFETs is revealed in Fig. 11e. The capacitance differences of NC-GAA FinFETs are larger than those of GAA FinFETs with respect to different numbers of HWKF. For NC-GAA FinFETs with the number of HWKF of 60, when the device is with |*C*_g,high_|, the values of polarization appearing at D, the middle of channel and S are − 6.3× 10^−7^, − 6.1 × 10^−7^ and − 7.2 × 10^−7^ C/cm^2^, respectively; additionally, when the device is with |*C*_g,low_|, they are − 3.6 × 10^−7^, − 3.5 × 10^−7^ and − 3.9 × 10^−7^ C/cm^2^. It implies that the different degree of polarization in the FE layer will be obtained due to the nonuniform electric field; thus, the variation of surface potential of NC-GAA FinFETs will become large. The *φ*_s_ (which is define by |*φ*_s,D_| − |*φ*_s,S_|) comparison for N-type GAA and NC-GAA FinFETs is shown in Fig. 11f. The results indicate that NC-GAA FinFETs have higher *φ*_s_ compared with GAA FinFETs for different numbers of HWKF; thus, NC GAA-FinFETs are more sensitive when suffering from the WKF. Figure 11g shows the charge density distribution of N-type GAA and NC-GAA FinFETs for the number of HWKF of 60. The induced channel charges in the NC-GAA FinFETs is more than that of the GAA FinFETs, which results in a larger magnitude of *C*_g_.

**Fig. 12:**
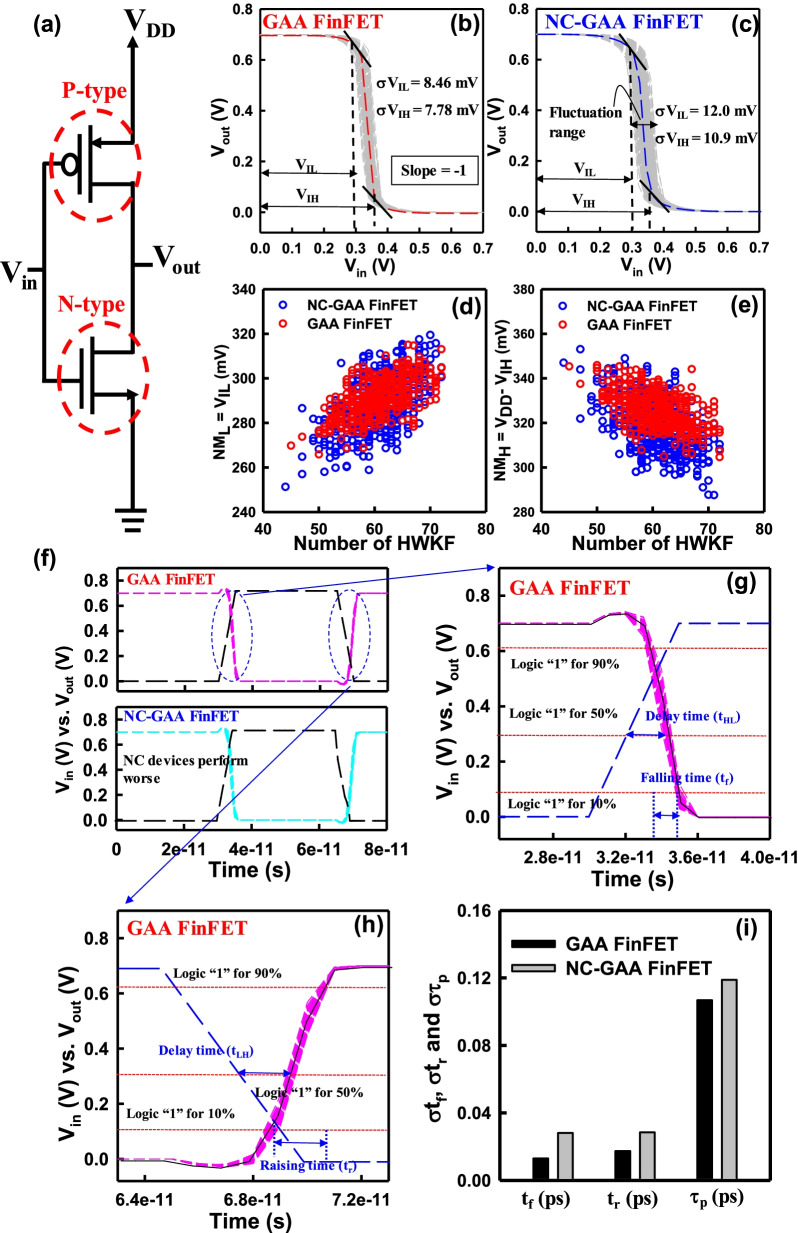
**a** The schematic plot of the inverter circuit. The voltage transfer curve for **b** GAA and **c** NC-GAA FinFETs. The NM_L_ and NM_H_ depend on *V*_IL_ and *V*_IH_ which are denoted in the plots. The comparison of NM_L_ and NM_H_ for **d** GAA and **e** NC-GAA FinFETs. **f** The *V*_in_ and *V*_out_ versus time for two different devices. The zoom-in plots which define the characteristics of **g** *t*_HL_ and *t*_f_ and **h** *t*_LH_ and *t*_r_ for GAA FinEETs. **i** The comparison of σt_f_, σt_r_ and στ_p_ for GAA and NC-GAA FinFETs

Based on the achieved DC and AC characteristics of GAA and NC-GAA FinFETs, we do further explore the behavior of inverter constructed by these devices. Figure 12a shows the schematic plot of the inverter circuit which is composed of a P-type transistor and a N-type transistor. The voltage transfer curves of GAA and NC-GAA FinFETs considering WKF are demonstrated in Fig. 12b, c. Due to the effect of WKF, NC-GAA FinFETs have a larger σ*V*_IL_ and σ*V*_IH_ [34, 35] than that of GAA FinFETs; the fluctuation of noise margin low (σNM_L_) of NC-GAA FinFETs is 12.0 mV and that of GAA FinFETs is 8.46 mV; the fluctuation of noise margin high (σNM_H_) of NC-GAA FinFETs is 10.9 mV and that of GAA FinFETs is 7.78 mV. The NM variation of NC-GAA FinFETs is with a 40%-increase compared with GAA FinFETs. Comparisons of NM_L_ and NM_H_ for GAA and NC-GAA FinFETs with respect to different HWKF numbers are shown in Fig. 12d, e, respectively. Figure 12f shows the *V*_in_ and *V*_out_ versus time for GAA and NC-GAA FinFETs. The zoom-in plot which defines the characteristic of high-to-low delay time (*t*_HL_), low-to-high delay time (*t*_LH_), falling time (*t*_f_) and raising time (*t*_r_) are shown in Fig. 12g, h. Figure 12i shows comparisons of σ*t*_f_, σ*t*_r_ and σ*τ*_p_ for GAA and NC-GAA FinFETs. Owing to different degrees of the FE polarization affected by the random position and number effects of WKF, the analyzed DC and AC characteristics including the results of *C*_g_ demonstrate that NC-GAA FinFETs have worse behavior than those of GAA FinFETs. Consequently, the performance including the NM and timing of the inverter with GAA FinFETs is superior to that of NC-GAA FinFETs.

## Conclusions

In summary, we have studied electrical characteristics of N-/P-type NC-GAA FinFETs for sub-3-nm technological nodes. The main findings of this work show that NC-GAA FinFETs have the better performance than that of GAA FinFETs. Compared with GAA FinFETs, more than 73% *I*_off_ suppression and 33% *I*_on_ boost have been achieved. Besides, we have analyzed the effect of WKF for 500 random devices; we observed that the *σV*_th_ of NC-GAA FinFETs is larger than that of GAA FinFETs, which increases the variability of N-type NC devices for 34% (((17.11 mV − 12.73 mV)/12.73 mV) × 100%) and P-type NC devices for 41% (((16.49 mV − 11.69 mV)/11.69 mV) × 100%). Based on the achieved DC and AC characteristics, the application of inverter with NC-GAA FinFETs has shown a worse performance including NM and timing than that of GAA FinFETs. The NM variation of NC-GAA FinFETs increases 40% compared with GAA FinFETs. This work has reported a comprehensive study for the emerging GAA FinFETs from the perspective of electrical and physical characteristics. It can benefit the device design and fabrication for sub-3-nm technological nodes.

## Data Availability

All data generated or analyzed during this study are included in this published article.

## Abbreviations

Si: Silicon
GAA: Gate-all-around
FinFET: Fin field effect transistor
NC: Negative capacitance
WKF: Workfunction fluctuation
SCE: Short-channel effect
WK: Workfunction
FE: Ferroelectric
*I*_on_: On-state current
HZO: HfZrO_*x*_
NCFET: NC field-effect transistors
SS: Subthreshold swing
*I*_off_: Off-state current
Si: Silicon
TiN: Titanium nitride
*V*_th_: Threshold voltage
3D: Three dimensional
S/D: Source/drain
ILD: Inter layer dielectric
*L*_g_: Gate length
*W*: Width
*L*_s_/*L*_d_: Source and drain extensions
EOT: The effective oxide thickness
H: Fin heights
LK: Landau–Khalatnikov
HWKF: High work function fluctuation
LWKF: Low work function fluctuation
MGN: Metal grain number
*G*: The average of grain size
*W*_eff_: Metal gate effective width
*C*_g_: The total capacitance
τ_P_: The delay time
*σV*_th_: The standard deviation of V_th_
σNM_L_: The standard deviation of noise margin low
*t*_HL_: The high-to-low delay time
*t*_LH_: The low-to-high delay time
*t*_f_: The falling time
*t*_r_: The raising time
